# Effect of handedness on brain activity patterns and effective connectivity network during the semantic task of Chinese characters

**DOI:** 10.1038/srep18262

**Published:** 2015-12-15

**Authors:** Qing Gao, Junping Wang, Chunshui Yu, Huafu Chen

**Affiliations:** 1 School of Mathematical Sciences, University of Electronic Science and Technology of China, Chengdu, 611731, China; 2 Department of Radiology, Tianjin Medical University General Hospital, Tianjin, 300052, China; 3 Key laboratory for Neuroinformation of Ministry of Education, School of Life Science and Technology, University of Electronic Science and Technology of China, Chengdu, 610054, China

## Abstract

Increasing efforts have been denoted to elucidating the effective connectivity (EC) among brain regions recruited by certain language task; however, it remains unclear the impact of handedness on the EC network underlying language processing. In particularly, this has not been investigated in Chinese language, which shows several differences from alphabetic language. This study thereby explored the functional activity patterns and the EC network during a Chinese semantic task based on functional MRI data of healthy left handers (LH) and right handers (RH). We found that RH presented a left lateralized activity pattern in cerebral cortex and a right lateralized pattern in cerebellum; while LH were less lateralized than RH in both cerebral and cerebellar areas. The conditional Granger causality method in deconvolved BOLD level further demonstrated more interhemispheric directional connections in LH than RH group, suggesting better bihemispheric coordination and increased interhemispheric communication in LH. Furthermore, we found significantly increased EC from right middle occipital gyrus to bilateral insula (INS) while decreased EC from left INS to left precentral gyrus in LH group comparing to RH group, implying that handedness may differentiate the causal relationship of information processing in integration of visual-spatial analysis and semantic word retrieval of Chinese characters.

Language and handedness are the most prominent lateralized cerebral functions, and the association of language lateralization and handedness has become the subject of intense research^1^,^2^,^3^. Handedness might be expected to provide an indicator of cerebral hemispheric language dominance^4^. Recent task-related neuroimaging studies on English language have consistently demonstrated that most of right-handed people exhibit a strong language lateralization in the left hemisphere; while left-handed individuals are believed to have a higher incidence of right dominant or bilaterally organized language representation^1^,^5^,^6^,^7^,^8^,^9^,^10^. These studies revealed regionally functional asymmetries of language involved brain regions associated with handedness during certain English language tasks in brain language areas, which were distributed in the frontal, temporal and parietal lobe^5^,^6^,^8^,^11^,^12^,^13^,^14^,^15^.

Recently, there is a great concern for integration and interaction of distributed neural systems for certain brain functions. Specifically, a growing emphasis has been placed on studies investigating the so called effective connectivity networks during language processing^16^. These studies suggested the cortical connectivity dynamics during certain language tasks^14^,^17^,^18^,^19^. Although the influence of handedness on the lateral brain activity patterns of language processes has been studied extensively in alphabetic languages, it remains unclear how the effective connectivity networks alter associated with handedness during language processing. In the semantic decision tasks, one study demonstrated right handers (RH) had higher modulations than left handers (LH) on the intrahemispheric connection on verbal stimuli; while LH had stronger modulations than RH on the intrahemispheric connection on nonverbal stimuli using dynamic causal modeling (DCM)^14^. Another study also applied DCM to a word production task data, and found LH showed significantly stronger connections originating in right fusiform gyrus (FG) to bilateral Brodmann’s area 44 comparing to RH^5^.

Unlike the linear structure of alphabetic words, Chinese characters have a square, nonlinear configuration, often having their meaning suggested by visual shapes^20^. These discrepancies in linguistic features lead to differential neural representations for Chinese and English processing. Neuroimaging studies have highlighted differences in neural systems for semantic processing of Chinese and alphabetic languages. For example, Studies on English semantic judgment experiments consistently found that the inferior frontal gyrus, the middle and inferior temporal gyri, and the fusiform gyrus were activated^14^,^21^,^22^,^23^; while in Chinese semantic tasks, additional engagement of the middle frontal gyrus (MFG) and the ventral occipito-temporal regions were found^20^,^21^,^24^,^25^,^26^. This may result in differential brain activity patterns of language lateralization associated with handedness. Although the influence of handedness on the laterality of language processes has been studied extensively in alphabetic languages, to our knowledge, few researches have been done on the activated brain regions specific to language lateralization associated with handedness during Chinese language processing. To take into account both LH and RH subjects would help to explain the probability and mechanism of atypical brain lateralization and handedness in character language cognition^27^. Concerning the effective connectivity network, to our knowledge few researches have been done to investigate the effect of handedness on the effective connectivity network in Chinese language processing. Using dynamic causal modeling, Xu *et al.* demonstrated information flowing from the visual cortex to left ventral occipito-temporal cortex to the parietal lobule and then to left MFG in a phonology based tone judgment task^17^. The interaction between left posterior middle temporal gyrus and left premotor cortex, and the causal influence from left posterior middle temporal gyrus to left primary motor cortex were found by Granger causality (GC) analysis during an action verb comprehension task^28^. These studies focused on the effective connectivity among relatively small number of regions of interest (ROI) only in RH group, thereby lacked investigation of the large-scale connectivity circuit among multiple regions involved in Chinese language processing, and the handedness influence on the effective connectivity language network.

Semantic access, the process of extracting meanings from different forms such as visually displayed words, plays a critical role in language comprehension^29^. However, it remains largely unknown how the neural systems dynamically interact with one another to support Chinese semantic processing. Neuroimaging studies suggest hierarchic coding of characters in the visual word semantic processing^29^. The visual stimuli firstly activate the occipital cortex, where the primary encoding of the visual stimuli is processed^29^. Then, the visual words map to a lexicon evoking the activation of evokes parts of temproparietal and parietal cortices, which are presumed a role in mapping visually presented inputs to linguistic representations^20^,^29^. Finally, the semantic access and integration are completed in regions such as temporal, parietal and frontal cortices, especially MFG for Chinese word processing^21^,^24^,^25^,^29^. It is essential and necessary to investigate the neural networks underpinning word semantic access using effective connectivity analysis. Furthermore, examining the impact of handedness on the Chinese semantic processing brain network could reveal novel aspects of brain lateralization^5^.

In the present study, aiming to investigate how handedness affects the activity patterns of language lateralization in native Chinese, and to inspect the dynamical interactions among the activated regions during Chinese semantic processing in LH and RH, the semantic task of Chinese characters in a blocked functional magnetic resonance imaging (fMRI) design was performed. The semantic decision task was used since it had demonstrated good agreement with previously standard, invasive methodologies and generated consistent strongly left-lateralized activation in brain for RH compared with phonological tasks^30^,^31^. Semantic task was also suggested to reduce the possible gender effect on language lateralization^32^. In our study, we focused on the overlapped activated regions in LH and RH groups, to ensure that the signal change of same regions and the connectivity among them were evaluated and compared. We hypothesized that

1. The lateralization of activity patterns during the semantic task of Chinese characters, including the signal changes in the conjoined activated areas is affected by handedness;
2. The effective connectivity networks among the overlapped activated regions recruited by Chinese semantic processing differs between LH and RH groups.

To test hypothesis (1), We carefully examined the influence of handedness on language lateralization by analyzing signal changes in the conjoined activated areas of LH and RH groups during the task, based on conjunction analysis^33^. The GC analysis was conducted to investigate the causal relations among the activated areas to test hypothesis (2). To ensure that the connectivity among the same ROIs was evaluated and compared^34^, the overlapped parts of the activated areas in both LH and RH groups were also chosen in the GC analysis. More particularly, the conditional GC (CGC) method in deconvolved blood oxygen level dependent (BOLD) level^35^ was applied to distinguish the pseudocausal relationship for three or more time series^36^, and to overcome the confounding effect of hemodynamic response function (HRF) in BOLD fMRI data^35^. The difference of CGC networks between LH and RH during the task was detected using one-tailed two sample *t*-tests. The difference of the numbers of Inter- and intra-hemispheric connectivity between the LH and RH groups were also investigated. The information flow among the network nodes was further evaluated by the graph-theoretic method of the In-Out degrees^34^, to explore the cause or target roles of the network nodes in LH and RH semantic processing networks.

## Results

### Behavioral Data

**Figure 1:**
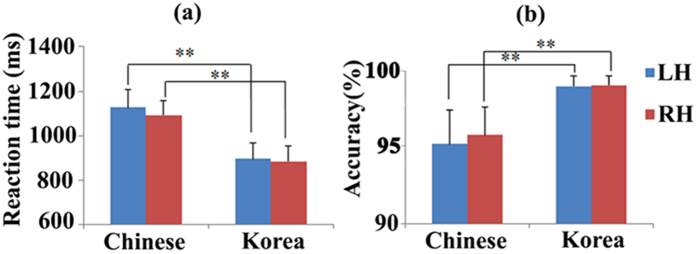
The behavioral performances in the two conditions. (**a**) Reaction time; (**b**) Accuracy. Error bars indicate standard deviation measurement. LH: Left handers; RH: Right handers. (**: significant threshold *p* < 0.01).

The mean Edinburgh Handedness Inventory (EHI) score for strong left handedness group was −66.4 ± 13.4, and was 96.8 ± 7.2 for strong right-handedness group. Reaction times for the semantic task (mean: 1128 ms for LH group and 1093 ms for RH group) were significantly longer than those for the control task (mean: 891 ms for LH group and 885 ms for RH group) (*p* < 0.01) in both LH and RH groups, whereas no significant difference was found between groups (Fig. 1a). Mean accuracies were 95.3% for the semantic task and 99.2% for the control task in LH group, and were 95.9% for the semantic task and 99.3% for the control task in RH group. The accuracies were significant lower (*p* < 0.01) for the semantic task compared with control task in both LH and RH groups (Fig. 1b). This suggested that the semantic decision task was more difficult than the visual word recognition task, and thereby ensured that the significantly activated areas would be considered to represent the main effect of Chinese meaning judgment. The between-group difference was not significant. The interaction effect was not significant, either.

### Brain activation during tasks

All the right-handed subjects had the lateralization index (LI) larger than 0.2 in the cerebral cortex (0.52 ± 0.10, range 0.32 ~ 0.71), indicating left-hemispheric language lateralization during the task in the cerebral cortex. 25 of the 28 left-handed subjects showed LI values between −0.2 to 0.2, indicating non-lateralized language activity pattern. 2 left-handed subjects had LI values larger than 0.2, while 1 left-handed subject had LI values lower than −0.2. The mean LI for all left-handed subjects was −0.02 ± 0.11 (range −0.22 ~ 0.21). Significantly different LI in the cerebral cortex was indicated between the two groups (*p* = 4e–26).

In the cerebellum, 27 of the 28 RH had LI lower than −0.2, while 1 indicated non-lateralized pattern with LI value of −0.19. The mean LI for all RH was −0.40 ± 0.10 (range −0.56 ~ −0.19). In the left-handed group, 24 of the 28 LH had LI values between −0.2 to 0.2, and 4 had LI lower than −0.2. The mean LI for all LH was −0.07 ± 0.11 (range −0.34 ~ 0.11). Significantly different LI in the cerebellum was also found between the two groups (*p* = 3e–16).

**Figure 2:**
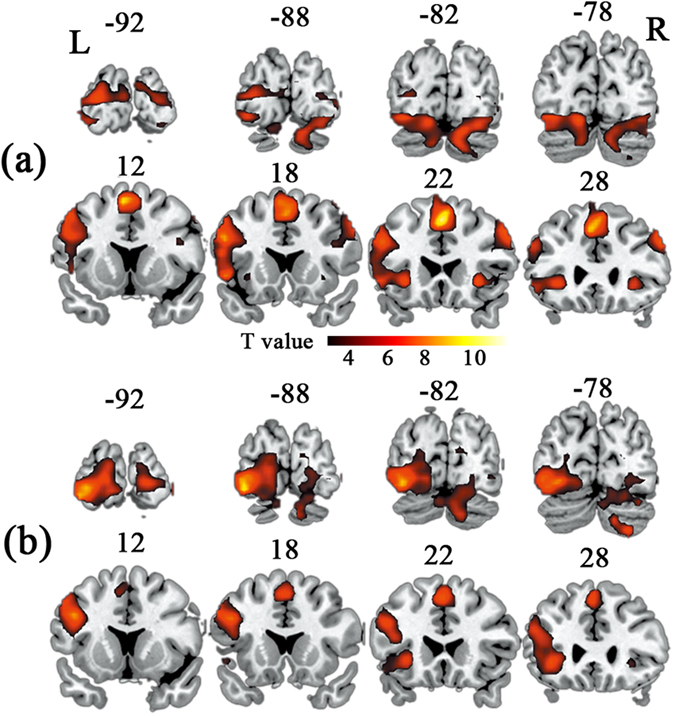
The significantly activated regions during the semantic task of LH (a) and RH (b) groups compared with the control task. The significant threshold is *p* < 0.01 FDR corrected, with cluster size >10.

Figure 2 shows the group analysis results of activated regions during the semantic task in LH (Fig. 2a) and RH (Fig. 2b) (false discovery rate (FDR) corrected for multiple comparisons, *p* < 0.01, and continuous cluster size > 10). The coordinates of peak voxels and statistical *t*-values for the LH and RH groups are reported in Table 1 and Table 2, respectively. The semantic task elicited stronger activations relative to the control task in distributed brain areas, encompassing inferior frontal gyrus (IFG), MFG, precentral gyrus (PreCG), supplementary motor area (SMA), insula (INS), occipital regions and cerebellum. More bilateral activity patterns in the frontal lobe, occipital lobe and cerebellum in LH group than those in RH group were visually demonstrated in Fig. 2. The locations of local maxima of significantly activated regions showed that RH exhibited an activated lateralization in the left hemisphere of cerebral cortex and in the right hemisphere of cerebellum (see Table 1 and Table2 for the details).

### Conjoint activated areas between LH and RH

**Figure 3:**
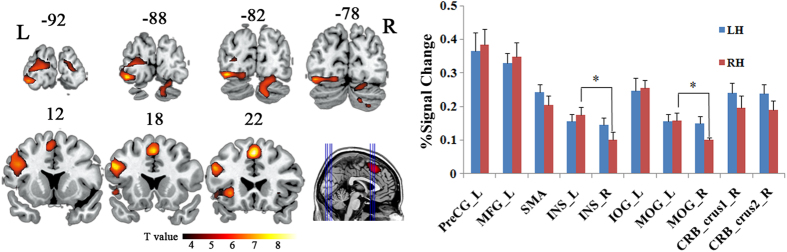
Conjoint activated areas during the semantic task in LH and RH groups (FDR corrected *p* < 0.01) along with the signal changes (%) in the conjoined activated areas. Error bars indicate standard deviation measurement. (*: significant threshold *p* < 0.05). CRB_crus1, cerebellum_crus1; CRB_crus2, cerebellum_crus2; INS, insula; IOG, inferior occipital gyrus; L, the left hemisphere; MFG, Middle frontal gyrus; MOG, Middle occipital gyrus; PreCG, precentral gyrus; R, the right hemisphere; SMA, supplementary motor area.

Table 3 demonstrates the details of the peaks of the conjoint activated areas during the task in LH and RH groups. Figure 3 depicts the conjoint activated areas in LH and RH groups, along with the signal changes (%) in the conjoint activated areas for LH and RH groups respectively. The conjoint activated areas included left MFG, left PreGC, SMA, bilateral INS, left inferior occipital gyrus (IOG), bilateral middle occipital gyrus (MOG) extending to FG and lingual gyrus, and right cerebellum_crus1 and _crus2. In the conjoint activated regions of the left hemisphere, RH showed higher signal changes comparing to LH; while in the regions of the right hemisphere, RH demonstrated lower signal changes comparing to LH, though the results did not reach statistical significance (See Fig. 3 for details). Significantly larger signal changes were found in left INS than that in right INS, and in left MOG than that in right MOG in RH group (*p* < 0.05). The conjunction analysis confirmed the observation of a left lateralization of activity pattern in RH group.

### Effective connectivity of the task in LH and RH

**Figure 4:**
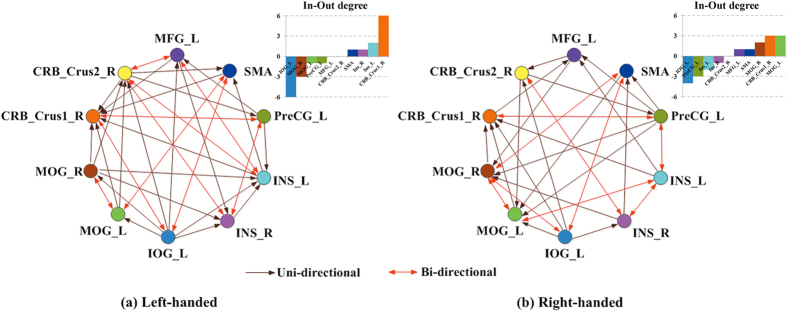
The networks of the statistically significant CGC components underlying the Chinese semantic task in LH and RH groups. (**a**) Results in LH group; (**b**) Results in RH group. Insets are the In-Out degrees of the nodes in each network. Abbreviations are the same as in Fig. 3.

Figure 4 demonstrates the networks of the statistically significant CGC components underlying the Chinese semantic task in LH (Fig. 4a) and RH (Fig. 4b), respectively. The brown arrow represents the unidirectional connectivity, while the red arrow represents the bidirectional connectivity. In both networks, the effective connections from the occipital to the temporal/parietal and then to the MFG were found. To further detect the difference of CGC networks between LH and RH during the task, one-tailed two sample *t*-tests were applied with the family-wise error (FWE) corrected threshold of *p* = 0.01. The significantly increased effective connections from right MOG to bilateral INS, and significantly decreased effective connection from left INS to left PreCG were found in LH group comparing to RH group.

The difference of the number of the inter-/intra-hemispheric connectivity between LH and RH groups was also investigated. The results demonstrated significantly more inter-hemispheric directional connections in LH than in RH (*p* < 0.01). There was no difference in the number of inter-hemispheric bidirectional connections between LH group and RH group (*p* = 0.06). Significantly different number of connections within the left hemisphere between LH and RH group was not detected (*p* = 0.64). Nor was the number of connections with in the right hemisphere (*p* = 0.07).

The figures of the In-Out degrees of the nodes in each network were inset in Fig. 4a,b, respectively. Regardless of handedness, left IOG had relatively high negative In-Out degrees, whereas right cerebellum_crus1 had relatively high positive In-Out degrees. This demonstrated that left IOG acted as causal source while right cerebellum_crus1 acted as causal target during the task for both LH and RH groups.

## Discussion

In the present study, we used the CGC method in deconvolved BOLD level to detect the effective connectivity network among the conjoint activated regions for LH and RH groups. Most of the BOLD-fMRI studies based on GCA always assumed homogeneous hemodynamic processes over the brain. However, the BOLD signal results from a coupling between the underlying neural activity and vascular hemodynamic responses^37^,^38^. Several studies have pointed out that HRF latency across distinct brain regions is variable, and the homogenous HRF assumption may disturb the inference of temporal precedence^35^,^37^,^39^,^40^. To investigate neuronal causal influences in different brain regions using GCA of BOLD-fMRI data should consider the confounding effect of HRF^35^,^38^,^41^. In order to overcome the issue, a novel CGC approach was proposed to reconstruct the HRF latency and deconvolved BOLD level effective connectivity network^35^. The study demonstrated deconvolution might remove spurious correlations and restore genuine correlations obscured by noise, and consequently increased the detection capacity of GCA of fMRI data to neural causality^35^.

The significantly activated areas were generated by the semantic task versus the control task, and were considered to represent the mainly effect of Chinese meaning judgment. Consistent with previous studies regarding Chinese semantic processing^20^,^21^,^24^,^25^,^42^,^43^, the semantic task elicited stronger activations relative to the control task in distributed area in the frontal gyrus, occipital regions and cerebellum. In the cerebral cortex, the RH group presented a left lateralized activity pattern, while the LH group presented more bilateral in activated regions than RH group (Table 1 and Table 2). The results were further demonstrated by the LI values of LH and RH. Our findings verified the ideas of bilateral speech representation in adextrals and possibly explained the results in the research by Carey and Johnstone, where they found dextrals and adextrals had similar dysphasia risk after left lesions, but adextrals increased risk after right lesions compared to dextrals^44^.

In cerebellum, our results showed activated areas to be right dominant in RH, which was contralateral to the activations of the cerebral cortex. Furthermore, in LH with bilateral activation in the prefrontal lobe and occipital lobe in the task, a more bilateral activity pattern was also found in cerebellum comparing to RH (Table 1 and Table 2). Cerebellar involvement in cognitive and linguistic processing has been verified by neuroimaging and clinical studies^42^,^45^,^46^,^47^,^48^,^49^,^50^,^51^. Specifically, language processing has found to be lateralized to right cerebellar hemisphere in RH with typical left hemisphere language dominance^42^,^51^,^52^,^53^; whereas in LH with right hemisphere language dominance a reversed pattern of language activations was found^54^,^55^. In keeping with these previous studies, our results further demonstrated a bilateral recruitment of both the cerebral and cerebellar language areas in LH during Chinese semantic processing^56^, and suggested the concept of a lateralized linguistic cerebellum associated with handedness^47^.

The more bilateral activity pattern in LH was further verified in the conjoint activated areas of LH and RH. Signal changes in the conjoint activated areas of the left hemisphere were found to be higher in RH than in LH; whereas in the conjoint activated areas of the right hemisphere, signal changes were lower in RH than in LH (Fig. 3). Even though the functional asymmetry differences did not reach significance, they informed us a more asymmetrical activation involvement even in conjoint activated areas in RH. This resulted in significantly different signal changes in bilaterally activated regions of INS and MOG in RH (Fig. 3). Specially, the activation lateralization in the two areas was associated with handedness in such a way that a left lateralization was presented in RH while a more bilateral pattern in LH, demonstrating cerebral representation of the word comprehension were divided more equally between the two hemispheres in LH^57^. One possible explanation may be related to callosal morphology: LH had been found to have a larger corpus callosum than RH^58^. This neuroanatomical difference between LH and RH was linked to the fact that LH had a more efficient exchange of information transmitted by callosal pathways^59^,^60^, resulting in better bihemispheric coordination and increased interhemispheric communication in LH. Our effective connectivity network results further demonstrated significantly more interhemispheric connections in LH group than in RH group during the task. The results suggested more information interchange occurred between the two hemispheres in LH during Chinese semantic processing. In brief, our findings gave direct evidence of a certain degree of bilaterality and increased interhemispheric communication of cerebral representation of language in LH. In addition, our findings partly explained the facts in patients with language disability: since additional recruitment of right hemisphere in LH was detected compared to RH, the disease may be associated with an increased rate of non-right-handedness and a trend towards brain symmetry^44^,^61^; increased interhemispheric communication in LH was also detected, which possibly compensated language function after the hemisphere injured and resulted in the facts that the severity of aphasia is milder in LH regardless of the hemisphere injured, and that the recovery is more rapid and more complete in these subjects than in RH^57^.

The CGC analysis also showed that in both networks, there were the circuits from occipital cortex to temporal/parietal cortex and then to MFG. Specifically, our results demonstrated that in the Chinese visual word semantic processing, information flowed from MOG and IOG to INS, then to PreCG, and then to MFG in the cerebral cortex. In addition, there were dense effective connections between these regions and right cerebellum_crus1 and _crus2, demonstrating intensive exchange of information between cerebellum and supratentorial areas. It has been proposed that the anatomically cerebello-cerebral cortical pathways provide a neural substrate for cerebellum to actively and directly participate in cognitive and linguistic processing^45^,^48^,^62^,^63^. Moreover, right cerebellum_crus1 consistently had relatively high positive In-Out degrees in the effective networks of both LH and RH groups, indicating that it acted as causal target during the Chinese semantic task for both LH and RH groups. Similar to the present study, a recent meta-analysis has also highlighted the roles of cerebellum_crus1 in language tasks by finding that one of the strongest activation peaks for the language tasks was located in right cerebellum_crus1^49^. In their study, they mentioned that anatomical and physiological studies in cats and non-human primates had demonstrated that association areas in both parietal and prefrontal cortical areas and crus1/crus2 were interconnected^49^,^64^. Our results further verified the important role of cerebellum_crus1 as an information receiver in the route of the effective connectivity network during the Chinese semantic processing. The results suggested that cerebellum_crus1 may be added in the language circuit as an essential node of Chinese semantic processing.

Furthermore, the present study demonstrated that bilateral MOG and INS were both recruited during the task in LH and RH group. Comparing to RH, LH demonstrated increased effective connections from right MOG to bilateral INS; while demonstrated decreased effective connection from left INS to left PreCG. INS was consistently found to be activated in Chinese language processing and was considered to be critical to language production and to correspond to a semantic word retrieval route^20^,^26^,^65^,^66^. INS has rich connectivity to both sensory processing regions and self-processing multimodal regions, suggesting a unique role for INS in the bottom-up and top-down information processing^67^,^68^. MOG together with FG and lingual gyrus consisted of the visual ventral route^29^. This ventral occipito-temporal system was believed to be associated with the primary visual coding for the present visual word and the word form pattern extracting for the later lexical and semantic access^29^. Therefore, our results pointed to the possible effects of handedness on the language causal network of the brain. We believed that LH group had stronger information transfer from right MOG to bilateral INS to better process visuo-spatial information before it reached the frontal gyrus. Handedness may differentiate the causal relationship of information processing in integration of visuo-spatial properties of Chinese character structures and semantic word retrieval of Chinese during the task.

Note that the absolute value of the mean EHI score for LH group was 66.4, which is much lower than that for RH group (96.8). Other than RH group who has the maximum EHI score of +100 which indicates exclusive right-handedness, LH group has the minimum EHI score of −80 instead of −100. All the left-handed subjects recruited in our study reported to use their right hands to write and eat. The reason is that in Chinese population, social pressure for right-handed writing and eating is very strong^69^,^70^. The born left-handed people are always forced to write and eat using their right hands^69^,^70^. Though this may bias the results in our study, our results still have universal meaning in Chinese population for the phenomena is very general in China.

## Conclusions

Using fMRI data, this study explored the brain activations of language lateralization and the causal network architecture among the conjoint activated areas of LH and RH during a Chinese semantic task. The results showed RH activated left lateralized in cerebral cortex and right lateralized in cerebellum; while LH were less lateralized than RH in both cerebral and cerebellar areas. Effective connectivity network analysis further demonstrated more interhemispheric connections in LH group than in RH group during the task, suggesting better bihemispheric coordination and increased interhemispheric communication in LH. Moreover, right cerebellum_crus1 was found to consistently act as causal target during the Chinese semantic task for both LH and RH groups, indicating cerebellum_crus1 as an essential node of the language circuit during the Chinese semantic processing. The effective connectivity analysis also suggested that handedness may differentiate the causal relationship of information processing in integration of visuo-spatial analysis and semantic word retrieval of Chinese characters. The new findings highlighted that handedness affected both brain activity patterns and effective connectivity network during the semantic task of Chinese characters, and might offer more detailed information about the mechanism underlying language lateralization and handedness.

## Methods

### Subjects

Thirty-four healthy left-handed subjects (seventeen females, age = 24.2 ± 2.5 years, range 19 ~ 29 years) and thirty-six healthy right-handed subjects (eighteen females, age = 24.7 ± 1.9 years, range 19 ~ 30 years) participated in the study after signing informed consents. All subjects were college students, with no history of psychiatric or neurological illness, or any impairment of function or language capability, and with normal or corrected to normal vision. Handedness was evaluated by the EHI^71^. Strong LH and RH were chosen to exclude ambidextrous participants. The criterion for strong left-handedness was an EHI score lower than −50, while the criterion for strong right-handedness was an EHI score larger than 50. Six left-handed and eight right-handed subjects were excluded from the criteria, leaving twenty-eight left-handed subjects (thirteen females, age = 24.2 ± 2.3 years, range 19 ~ 27 years) and twenty-eight right-handed subjects (fourteen females, age = 24.5 ± 1.8 years, range 20 ~ 29 years) for further study. All the subjects were native Chinese speakers and reported no exposure to Korean, to ensure that Korean characters would serve as an appropriate perceptual control task. The present study protocol was approved by the local Ethics Committee of Tianjin Medical University and was carried out in accordance with the approved guidelines.

### Experimental Paradigm

The experiment was performed on a 3.0-T GE Signa HDx MR scanner (Tianjin medical university, Tianjin, China) using a gradient-recalled echo planar imaging (EPI) sequence with an 8-channel head coil. The acquisition parameters for functional imaging were as follows: TR = 3000 ms, TE = 30 ms, FOV = 22 cm, matrix = 64 × 64, voxel size = 3.44 × 3.44 × 4 mm^3^, 38 transverse slices with slice thickness = 3 mm, slice gap = 1 mm, and flip angle = 90°. High-resolution 3D T1-weighted anatomical images were also acquired in sagittal orientation using a fast spoiled gradient recalled sequence (BRAVO, TR = 7.8 ms, TE = 3.0 ms, flip angle = 7°, matrix size = 256 × 256 × 176, slice thickness = 1 mm without slice gap, and voxel size = 1 × 1 × 1 mm^3^).

During the active condition, participants were asked to judge if two Chinese characters appeared on the screen had the same meaning. The targets and foils were randomly chosen with the ration of 1:1. During the control condition, the participants were asked to judge whether the two Korean characters were the same or not. The Korean characters were also randomly chosen with half of them the same and half of them different. In the experiment, Korean was chosen because it is similar to Chinese characters in terms of visual complexity and configuration^72^. The participants had practiced a short version of the experimental task to become familiar with the tasks. If the subject’s accuracy rate was higher than 80% during the practice, he/she was allowed to participate in the fMRI experiment.

**Figure 5:**
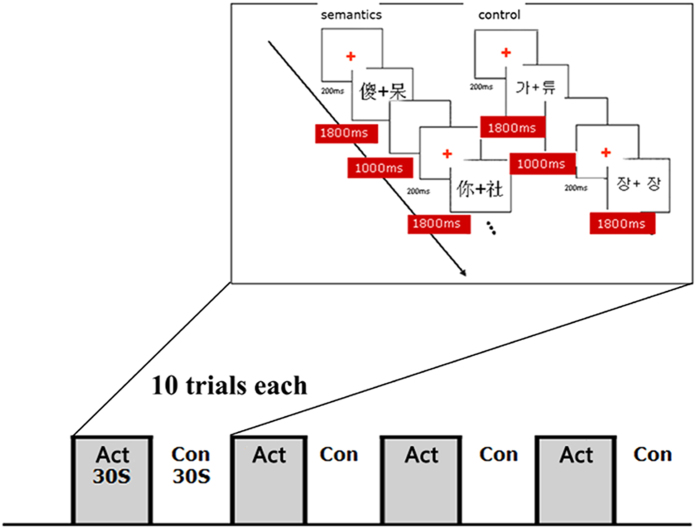
The diagram of experimental design. 4 semantic task blocks and 4 control task blocks were designed alternatively, and each block consisted of 10 trials lasting 30 seconds.

The fMRI experiment was a blocked design, with 4 semantic task blocks and 4 control task blocks alternatively. Each block lasted 30 seconds with 10 trials. On each trial, firstly the “+” cue was presented in the center of the screen for 200 ms, then two Chinese characters was visually displayed for 1800 ms in the semantic condition (or two Korean characters in the control condition), followed by a 1000 ms blank interval for the subjects to press the key. Figure 5 demonstrates the diagram of the experimental design. The visual angles were 16.5° in length and 11.8° in width, and the size of the stimuli was 10.0 × 4.5 mm^3^. Participants gave a positive response by pressing the key corresponding to the index finger of their right hand; while a negative response by pressing the key corresponding to the index finger of their left hand. Participants were asked to perform the tasks as quickly and accurately as possible.

### Data Analyses

The acquired images were preprocessed using statistical parametric mapping (SPM) software (SPM8, http://www.fil.ion.ucl.ac.uk/spm). The first five images were dummy scans and thereby were discarded for scanner stabilization. The remaining 80 images were firstly corrected for the acquisition time delay among different slices, and then were realigned onto the first image for head-motion correction. The dataset with translational or rotational parameters exceeding ±1 mm or ±1° would be excluded. No dataset was excluded by the criteria. The images were then spatially normalized into a standard stereotaxic space with voxel size of 3 × 3 × 3 mm^3^ using the Montreal Neurological Institute (MNI) EPI template.

The statistical parametric maps (*t*-statistics) of contrasting between the semantic condition and the control condition were generated by using the general linear model. A group analysis was performed with a threshold at *p* < 0.01 (FDR corrected for multiple comparisons), and an extent threshold of 10 contiguous voxels. Conjunction analysis was performed using SPM8 to obtain the conjoint activated areas of LH and RH groups^33^, with a threshold of *p* < 0.01 (FDR corrected). Functional regions of interest (ROI) were generated by masking the thresholded group conjoint activated map with the automated anatomical labeling (AAL) template to further analyze the signal changes in each ROI. The representative time series in each ROI was obtained by averaging the fMRI time series across all voxels in the ROI. Signal change of each ROI was calculated using the MarsBaR toolbox (http://www.sourceforge.net/projects/marsbar).

### Lateralization index assessment

To assess the language lateralization, the threshold-free lateralization index (LI) on SPM t-maps was calculated for each subject using the approach proposed by Nagata *et al.*, which could minimize the influence of the statistical threshold on LI assessment^73^,^74^. This approach calculates the number of left and right hemisphere voxels activated for the task relative to the baseline, at a range of different statistical thresholds, then searches for the best regression function describing the relationship between the number of voxels and the statistical threshold^13^,^73^. The regression provides a constant term that is used to compute a normalized difference between left and right hemisphere activity^13^,^73^. A positive value of LI represents left-hemisphere dominance, whereas a negative value indicates right-hemisphere dominance^14^. We calculated LI for the cerebral cortex and the cerebellum, respectively. As previous studies suggested, language lateralization was tabulated based on the following categories: right-lateralized (LI ≤ −0.2), left-lateralized (LI ≥ 0.2), non-lateralized (−0.2 < LI < 0.2)^30^.

### Conditional Granger causality analysis

In the current study, the CGC method in deconvolved BOLD level for effective connectivity analysis was performed among the selected ROIs using an in-house MATLAB toolbox (http://guorongwu.weebly.com/software.html). The HRFs for deconvolution were obtained by modeling signal dynamics with the task inputs^35^. The causal influence was calculated for ROIs of each subject. Subsequently, the nonparametric bootstrap methodology was applied to assess the statistically significant threshold of the CGC components among the regions^34^. In addition, the In-degree and Out-degree of the nodes in CGC causal connectivity networks were calculated for each group to evaluate the causal in/out flow connections of each node in the CGC network^75^. The In-Out degrees of the nodes, which were defined as the difference between each node’s In-degree and Out-degree, were then sorted in an ascending order to identify causal target or causal source level^75^. If the In-Out degrees are the same for two nodes, the order was further sorted by the descending order of their Out-degree if the In-Out degrees <0 or by the ascending order of their In-degree if the In-Out degrees ≥ 0^34^. Afterward, estimated GC values of each paired ROIs were compared for the two groups using one-tailed two-sample *t*-tests (the FWE corrected threshold of *p* = 0.01), to detect the difference of the effective connectivity networks between LH and RH groups. Finally, the difference of the number of the inter-/intra-hemispheric connectivity between the LH and RH groups were also investigated using two sample *t*-tests (*p* < 0.01). The statistic tests were performed to test 1) whether there are more inter-hemispheric directional connections in LH group than in RH group; 2) whether there are more inter-hemispheric bidirectional connections in LH than in RH group; 3) whether the number of connections within the left hemisphere is different in LH than in RH group; 4) whether the number of connections within the right hemisphere is different in LH than in RH group.

## Additional Information

**How to cite this article**: Gao, Q. *et al.* Effect of handedness on brain activity patterns and effective connectivity network during the semantic task of Chinese characters. *Sci. Rep.* **5**, 18262; doi: 10.1038/srep18262 (2015).

## Figures and Tables

**Table 1:** Local maxima of significantly activated regions in LH group.

Region name	Abbreviation	Hem	Coordinates			Peak *t*-value
			X	Y	Z	
Frontal						
Inferior frontal gyrus, orbital	IFGorb	L	−45	39	−3	7.89
		R	45	42	−6	3.78
Inferior frontal gyrus, triangular	IFGtri	L	−54	18	15	6.25
		R	57	24	24	4.85
Middle frontal gyrus	MFG	L	−51	21	33	8.78
		R	54	27	33	8.29
Parietal-(pre)Motor						
Precentral gyrus	PreCG	L	−45	3	54	7.01
Supplementary motor area	SMA	L/R	0	24	48	11.33
Occipital						
Inferior occipital gyrus	IOG	L	−36	−81	−12	7.56
		R	32	−84	−14	6.10
Middle occipital gyrus	MOG	L	−24	−99	9	7.93
		R	27	−96	6	6.13
Superior occipital gyrus	SOG	L	−12	−102	18	7.43
		R	15	−99	18	6.96
Paralimbic						
Insula	INS	L	−30	24	−3	7.66
		R	30	24	−3	7.76
Cerebellum						
Cerebellum_6	CRB_6	L	−15	−78	−15	7.92
		R	30	−69	−24	4.70
Cerebellum_crus1	CRB_crus1	R	15	−81	−24	7.27
Cerebellum_crus2	CRB_crus2	L	−12	−78	−33	5.68
		R	12	−81	−33	6.89

**Table 2:** Local maxima of significantly activated regions in RH group.

Region name	Abbreviation	Hem	Coordinates			Peak*t*-value
			X	Y	Z	
Frontal						
Inferior frontal gyrus, triangular	IFGtri	L	−51	30	24	6.57
Inferior frontal gyrus, orbital	IFGorb	L	−51	18	−6	4.73
Middle frontal gyrus	MFG	L	−48	15	42	4.96
Parietal-(pre)Motor						
Precentral gyrus	PreCG	L	−45	9	33	8.96
Supplemetary motor area	SMA	L	−3	24	48	7.78
Occipital						
Calcarine		L	−9	−96	−6	6.69
		R	21	−96	0	7.06
Lingual gyrus	LING	L	−15	−72	−9	5.78
Inferior occipital gyrus	IOG	L	−36	−90	−9	10.33
Middle occipital gyrus	MOG	L	−15	−99	3	7.99
Paralimbic						
Insula	INS	L	−33	24	−3	7.89
		R	30	27	−3	4.68
Cerebellum						
Cerebellum_6	CRB_6	R	24	−75	−21	5.24
Cerebellum_crus1	CRB_crus1	R	36	−72	−27	5.89
Cerebellum_7b	CRB_7b	R	27	−75	−48	7.34
Cerebellum_crus2	CRB_crus2	R	15	−78	−39	6.56

**Table 3:** Conjunction analysis of significantly activated regions during the semantic task of LH and RH groups.

Region name	Abbreviation	Hem	Coordinates			Peak *t*-value
			X	Y	Z	
Frontal						
Middle frontal gyrus	MFG	L	−51	18	36	8.10
Parietal-(pre)Motor						
Supplementary motor area	SMA	L/R	0	24	48	8.62
Precentral gyrus	PreCG	L	−51	9	45	7.31
Paralimbic						
Insula	INS	L	−33	24	−3	7.19
		R	30	24	−3	4.99
Occipital						
Inferior occipital gyrus	IOG	L	−36	−84	−12	8.01
Middle occipital gyrus	MOG	L	−18	−99	9	6.20
		R	24	−96	6	5.43
Cerebellum						
Cerebellum_crus1	CRB_crus1	R	18	−84	−21	5.61
Cerebellum_crus2	CRB_crus2	R	9	−84	−33	6.19
